# Supplementary material for: Pangolin-inspired untethered magnetic robot for on-demand biomedical heating applications
Source: Nat Commun. 2023 Jun 20;14:3320. doi: 10.1038/s41467-023-38689-x (PMC10282021; doi:10.1038/s41467-023-38689-x)
Supplement: Supplementary file 1 — Supplementary Information [file 41467_2023_38689_MOESM1_ESM.pdf]

Supplementary Information for

Pangolin-inspired untethered magnetic robot for on-demand biomedical heating applications

Ren Hao Soon[1,2†], Zhen Yin[1†], Metin Alp Dogan[1], Nihal Olcay Dogan[1,2], Mehmet Efe Tiryaki[1,2], Alp Can Karacakol[1], Aslı Aydın[1], Pouria Esmaeili-Dokht[1] and Metin Sitti[1,2,3*]

[1] Physical Intelligence Department, Max Planck Institute for Intelligent Systems, 70569 Stuttgart, Germany
[2] Institute for Biomedical Engineering, ETH Zürich, 8092 Zürich, Switzerland
[3] School of Medicine and College of Engineering, Koç University, 34450 Istanbul, Turkey

[†] Equally contributing first authors
* Correspondence to: sitti@is.mpg.de

This PDF file includes:

Supplementary Text
Supplementary Figures 1 to 12
Supplementary Tables 1 to 2
Codes: Data Processing
Supplementary References

Supplementary Text

1. Analysis of the system

Governing equation (per unit time)

Assuming no contributions from magnetic hysteresis, $T_{ambient}$ = 25°C,

$$P_{in} = mc_p \frac{\partial T}{\partial t} + H_L$$

$$P_{in} = \rho V c_p \frac{\partial T}{\partial t} + H_L \qquad (1)$$

where P_{in} = input power,
m = mass of the material in kg ,
ρ = density of the material in kg m^{-3},
V = volume of the material in m^3,
c_p = specific heat capacity at constant pressure of the material in J kg^{-1} K^{-1},
$\frac{\partial T}{\partial t}$ = rate of change of temperature in K s^{-1},
H_L = heat losses in W.

Heat generated (*1, 2*)

From Faraday's law, the change in magnetic field, $\frac{\partial \mathbf{B}}{\partial t}$, induces a potential difference, $\mathbf{E}$, within the material of the following magnitude:

Since the plates are sufficiently far away (5 cm) and thin (< 300 μm), $\frac{\partial \mathbf{B}_{\mathbf{applied}}}{\partial t}$ is assumed to be constant and homogenous.

Note: $\mathbf{B}$ does not necessarily refer to the $\mathbf{B}_{\mathbf{applied}}$ because $\mathbf{B}$ decays as it travel through the material (i.e. current preceding layers reduce the magnitude of $\mathbf{B}$ in the current layer).

$$\nabla \times \mathbf{E} = -\frac{\partial \mathbf{B}}{\partial t}$$

Since $\nabla . \mathbf{B} = 0, \mathbf{B} = \nabla \times \mathbf{A}$, where $\mathbf{A}$ is the magnetic vector potential,

$$\Rightarrow \frac{\partial \mathbf{B}}{\partial t} = \nabla \times \frac{\partial \mathbf{A}}{\partial t}$$

$$\therefore \ \nabla \times \mathbf{E} = -\nabla \times \frac{\partial \mathbf{A}}{\partial t}$$

$$\mathbf{E} = -\frac{\partial \mathbf{A}}{\partial t} - \nabla C$$

where C = electric scalar potential.

Assuming material is ohmic ($\mathbf{J} = \boldsymbol{\sigma}\mathbf{E}$),

$$\mathbf{J} = -\boldsymbol{\sigma}\frac{\partial \mathbf{A}}{\partial t} + \mathbf{J_s}$$

where $\mathbf{J_s} = -\boldsymbol{\sigma}\,\nabla C$ = current density in the source (i. e. induction coil).

∴ At constant input power from the RF coil $\mathbf{J_s}$, the induced current density, $\mathbf{J}$,

is directly proportional to the conductivity, $\boldsymbol{\sigma}$, which is an intensive property of the material.

For a thin layer where $\mathbf{B}$ can be assumed to be constant, $\mathrm{B_z}$, and assuming an isotropic material,

$$\dot{P}_{layer_z} = \iint \frac{\mathbf{J^2}}{\boldsymbol{\sigma}} dA \approx \frac{\left[-\frac{d(\mathrm{B}_z.A)}{dt}\right]^2}{\frac{\rho l}{0.5l}}$$

where A = area perpendicular to the applied field (i. e. $\mathrm{l}^2 -$ square plate).

$$\Rightarrow \dot{P}_{layer_z}\ \alpha\ \sigma$$

$$P_{in} = \int_{o}^{h} \dot{P}_{layer_z}\, dz\ \alpha\ \sigma$$

$$\Rightarrow P_{in}\ \alpha\ \sigma$$

Heat loss

Assuming incompressible flow and homogenous temperature throughout the scale,

Heat losses are caused by convective and radiative losses,

$$H_L = Convection + Radiation$$

$$= h_{top}A_{top}(T_s - T_{ambient}) + h_{side}A_{side}(T_s - T_{ambient}) + \sigma\epsilon A_{top\ and\ side}(T_S^4 - T_{ambient}^4)$$

where $\sigma = 5.67 \times 10^{-8}$ W m^{-2} K^{-4},
ϵ = emissivity of surface,
A = area in m^2,
$\mathrm{T_s}$ = current temp of surface in K,
$\mathrm{T_{ambient}}$ = assumed to be 298.15 K,
h = convective heat transfer coefficient in W m^{-2} K^{-1}.

To calculate the convective heat transfer coefficient h (3),

$$Ra = Gr_{l_c} Pr = \frac{\beta(T_s - T_{ambient}) l_c^3 g}{\eta \alpha}$$

where $\beta = 3.38 \times 10^{-3} K^{-1}$ (coefficient of thermal expansion at 25 °C),
$g = 9.81 \, m \, s^{-2}$,
l_c = characteristic length in m,
T_s = current temperature of surface in K,
$T_{ambient}$ = 298.15 K,
$\eta = 1.568 \times 10^{-5}$ Pa s (kinematic viscosity of air at 25 °C),
$\alpha = 19 \times 10^{-6} \, m^2 \, s^{-1}$ (thermal diffusivity of air at 25 °C).

For $Ra_L < 10^9$ and constant heat flux out of a vertical plate,

Assuming $Ra < 10^9 \because O(l_c) = O(w) = 10^{-6}$ m,

$$\therefore Nu = 0.68 + \frac{0.67 Ra^{1/4}}{\left[1 + \left(\frac{0.492}{Pr}\right)^{9/16}\right]^{4/9}}$$

For $Gr_L Pr < 2 \times 10^8$ and constant heat flux out of a horizontal plate (heated side up),

$$\therefore Nu = 0.13(Gr_L Pr)^{1/3} = 0.13 \, Ra_L^{1/3}$$

$$Ra < 10^9 \because O(l_c) = O(w) = 10^{-6} \, m$$

$$h = \frac{Nu.k}{L_c}$$

where $k = 26.38 \times 10^{-3} \, Wm^{-1}K^{-1}$ (thermal conductivity of air at 25 °C).

2. Influence of material properties on heating performance

The other material properties identified are namely the specific heat capacity at constant pressure c_p, thermal conductivity k, and density ρ. Since the effects of each of the factors could not be independently studied in real life, simulations were used to study the effects. The range of values used in the simulations were kept within the limits of conductive materials found in real life. At t = ∞, the final temperature was independent of the density (fig. S1B) and specific heat capacity (fig. S1C). The temperature of the scales in the simulations were independent of the thermal conductivity regardless of the time (fig. S1D and G). At t = 1, a lower density (fig. S1E) and specific heat capacity (fig. S1F) would result in a higher rate of temperature increase.

3. Influence of electrical conductivity on maximum temperature

This effect was observed in simulations where we tracked the changes in magnetic flux density through the centre of the material. Specifically, we observed that the magnetic field penetrated deeper into materials with lower electrical conductivities. For a 50 µm thick 1 cm^2 square sample with electrical conductivities of 1 x 10^6 S m^{-1} and 1 x 10^8 S m^{-1}, the magnetic flux density at the centre of the material (i.e. 25 µm) into the material was 1.62 mT and 0 mT, respectively (fig. S2B). The same trends were observed in a 250 µm thick material (fig. S2C). Since there was no magnetic flux density in the centre of the material as the electrical conductivity was increased, the induced currents were confined to a smaller region along the z-axis in materials with higher electrical conductivities.

4. Influence of electrical conductivity on temperature rise time

Looking at equation (1), the heat losses at t = 0 are zero since the metal will be at ambient temperature. As such, equation (1) can be reduced to $\frac{\partial T}{\partial t} = \frac{P_{in}}{\rho V c_p}$, implying that all of P_{in} will be used to heat the scale. Consequently, materials with a lower $\rho V c_p$ (i.e., lower mass, volume and/or a specific heat capacity) will register a higher $\frac{\partial T}{\partial t}$, temperature rise. From the experiments, it was observed that a plate with a smaller thickness (less *V*) was able to generate heat at a faster rate than a thicker plate (Fig. 2G, fig. S1H and fig. S1I). We also note that using a material with a lower density and/or the specific heat capacity as described by equation (1) would also result in a higher $\frac{\partial T}{\partial t}$ (fig. S1E and F).

The optimal ratios presented in Figure 2F are also independent of the input current (fig. S2F). Should a higher temperature be required with a smaller scale, a higher input RF current could be given to mitigate the decrease in heating performance arising from using a smaller scale. Moreover, since $\frac{1}{\delta xy}$ is also a function of the frequency, *f*, this implies that even if a more electrically conductive material is used, the same thickness can be used as long as the frequency is reduced to compensate for it.

5. Initial biocompatibility studies

Results from an initial biocompatibility test are presented here. In this regard, the aluminium scale was cultured with a human fibroblast cell line. After 72 h of culture, the fibroblasts demonstrated clear viability in the culture environment with spindle-shaped, healthy morphology at the interface of the metal (fig. S8A). Aluminium powder was also cultured with the fibroblasts at different concentrations. The cell viability analysis showed that the aluminium powder (up to the concentration of 600 µg mL^{-1}) did not cause any adverse effect on the cells after 72 hours (fig. S8B).

In addition, a 1 cm^2 aluminium scale of 100 µm thickness was submerged in 2 mL of simulated gastric fluid, intestinal fluid and high-glucose Dulbecco's Modified Eagle Medium (DMEM) to test the stability of the material over 48 h. No observable degradation occurred for the samples submerged in intestinal fluid and DMEM. The aluminium scale submerged in simulated gastric fluid dissolved after 48 h. The absorbance of the resultant solution was 0.367 which corresponded to a

concentration of 533.6 µg mL^{-1} (fig. S9). This preliminary data indicates that even if the aluminium scale dissolves, the concentration does not adversely affect cell viability.

6. Environmental impact on locomotion

The pangolin-inspired overlapping design introduced in this work utilises rolling for locomotion. In terms of how the environment would influence rolling, we refer to a publication (*4*), in which a rolling wheel (no slip) was found to be a good approximation for the robot's rolling at low frequencies (< 10 Hz). As such, the equation of motion for the rolling robot can be expressed as:

$$\mathbf{F_T} - \mathbf{F_A} - \mathbf{F_R} = m\ddot{\mathbf{x}} \tag{2}$$

where $\mathbf{F_T}$ is the traction force, $\mathbf{F_A}$ is the aerodynamic drag, $\mathbf{F_R}$ is the rolling resistance, m is the mass of the robot and $\ddot{\mathbf{x}}$ is the linear acceleration (fig. S10).

At steady state, $\ddot{\mathbf{x}} = 0$ and Eq. 2 reduces to:

$$\mathbf{F_T} = \mathbf{F_A} + \mathbf{F_R} \tag{3}$$

Next, we consider each term in Eq. 3 separately.

$\mathbf{F_T}$ can be re-expressed as:

$$\mathbf{F_T} = \frac{\boldsymbol{\tau}_{\mathbf{Roll}}}{r_{eff}}$$

where $\boldsymbol{\tau}_{\mathbf{Roll}}$ is the magnetic torque applied and r_{eff} is the effective radius.

$\mathbf{F_A}$ can be re-expressed as:

$$\mathbf{F_A} = \frac{1}{2}\rho A\mathbf{V^2}C_D$$

where ρ is the density of the fluid, A is the reference area, $\mathbf{V}$ is the velocity and C_D is the drag coefficient. In this work, A is the frontal area and can be taken to be the following:

$$A = 2r_{eff}.b$$

where b is the width of the robot.

On the other hand, C_D is sensitive to changes in Reynold's number (*Re*) and can vary by several orders of magnitude. To illustrate this, we consider the 20 x 10 x 0.2 mm robot used in this work. Assuming the robot's velocity to be 0.05 m/s, a characteristic length of 0.01 m, and the density of fluid to be 1000 kg/m^3 – the major component of most biological fluids is water and remains fairly constant (*5*), sweeping the dynamic viscosity from 10^{-3} to 10^2 (*6*) would result in a *Re* in the range of 10^{-3} to 10^2. Consequently, C_D can vary anywhere between 10^0 to 10^2 (*7*).

For the rolling resistance, $\mathbf{F_R}$, hysteresis losses constitute the bulk of it. For a wheel, these losses are dependent on the normal load, temperature, speed and contact friction (i.e. friction between the metal scale and tissue surface) (*8*).

Taken together, this implies that the contact friction and viscosity are the dominant environmental properties affecting magnetic actuation. When the viscosity is increased, the resultant increase in Re and C_D, means that $\mathbf{F_A}$ would increase correspondingly. This results in a lower $\ddot{\mathbf{x}}$ and V. Similarly, when the contact friction is increased, a lower speed would be achieved given the same inputs (i.e. actuating magnetic (B) field and magnetisation, M, of the robot).

The extent to which rolling is affected highly depends on how these parameters change and requires further studies for a number of reasons. Firstly, biological fluids are typically non-Newtonian and exhibit shear thinning behaviours (*6*). Moreover, the above analysis only holds true when the entire robot is submerged in the fluid. In certain scenarios, such as those presented in this work, the robot might only be partially submerged in the fluid (e.g. only the scales). Lastly, the scales in this work could potentially even enhance locomotion. In this regard, similar to how snow chains help wheels to grip the road and prevent skidding, these scales could also help the robot dig into a layer coated with mucus and therefore prevent the robot from slipping.

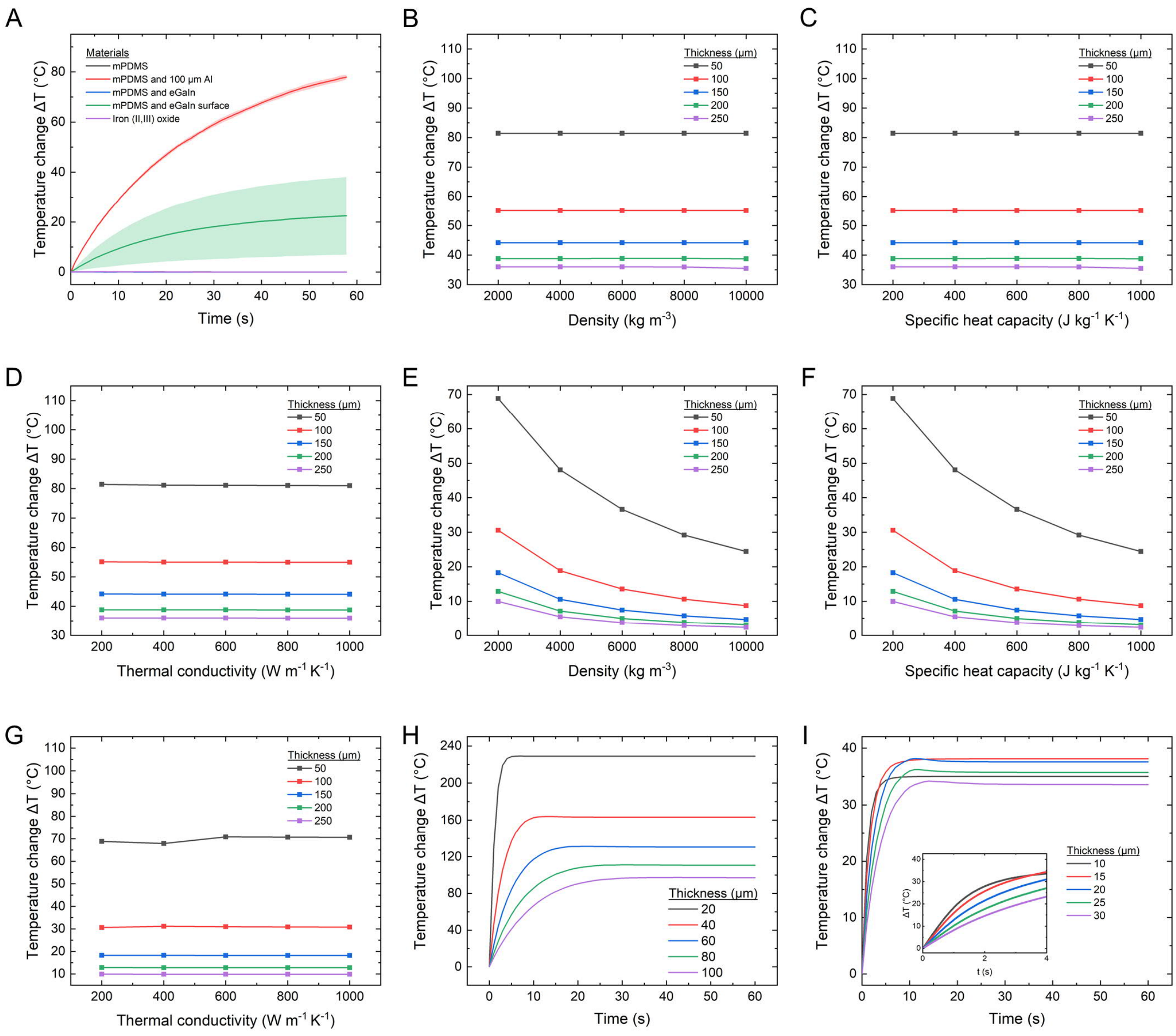

Supplementary Figure 1. Characterisation of the remote heating performance. (**A**) Experimental comparison of various methods of heat generation using a radio-frequency field at a distance of 5 cm (n = 6). All samples have a heating layer thickness of 100 µm and dimensions of 1 cm x 1 cm. As the samples made from mPDMS, mPDMS and eGaIn, and iron (**II**, **III**) oxide do not record any temperature change, the graphs of these samples overlap one another. (**B**) Simulated temperature change of a 100 µm aluminium scale with different densities at t = 60 s. (**C**) Simulated temperature change of a 100 µm aluminium scale with different specific heat capacities at t = 60 s. (**D**) Simulated temperature change of a 100 µm aluminium scale with different thermal conductivities at t = 60 s. (**E**) Simulated temperature change of a 100 µm aluminium scale with different densities at t = 1 s. (**F**) Simulated temperature change of a 100 µm aluminium scale with different specific heat capacities at t = 1 s. (**G**) Simulated temperature change of a 100 µm aluminium scale with different thermal conductivities at t = 1 s. (**H**) Simulated temperature change of aluminium scale with different thicknesses over time. (**I**) Simulated temperature change of aluminium scale with different thicknesses over time (low thicknesses). Note that the simulated temperatures presented in this figure are lower than those presented previously because the metal scales are modelled with the thin shell approximation in COMSOL.

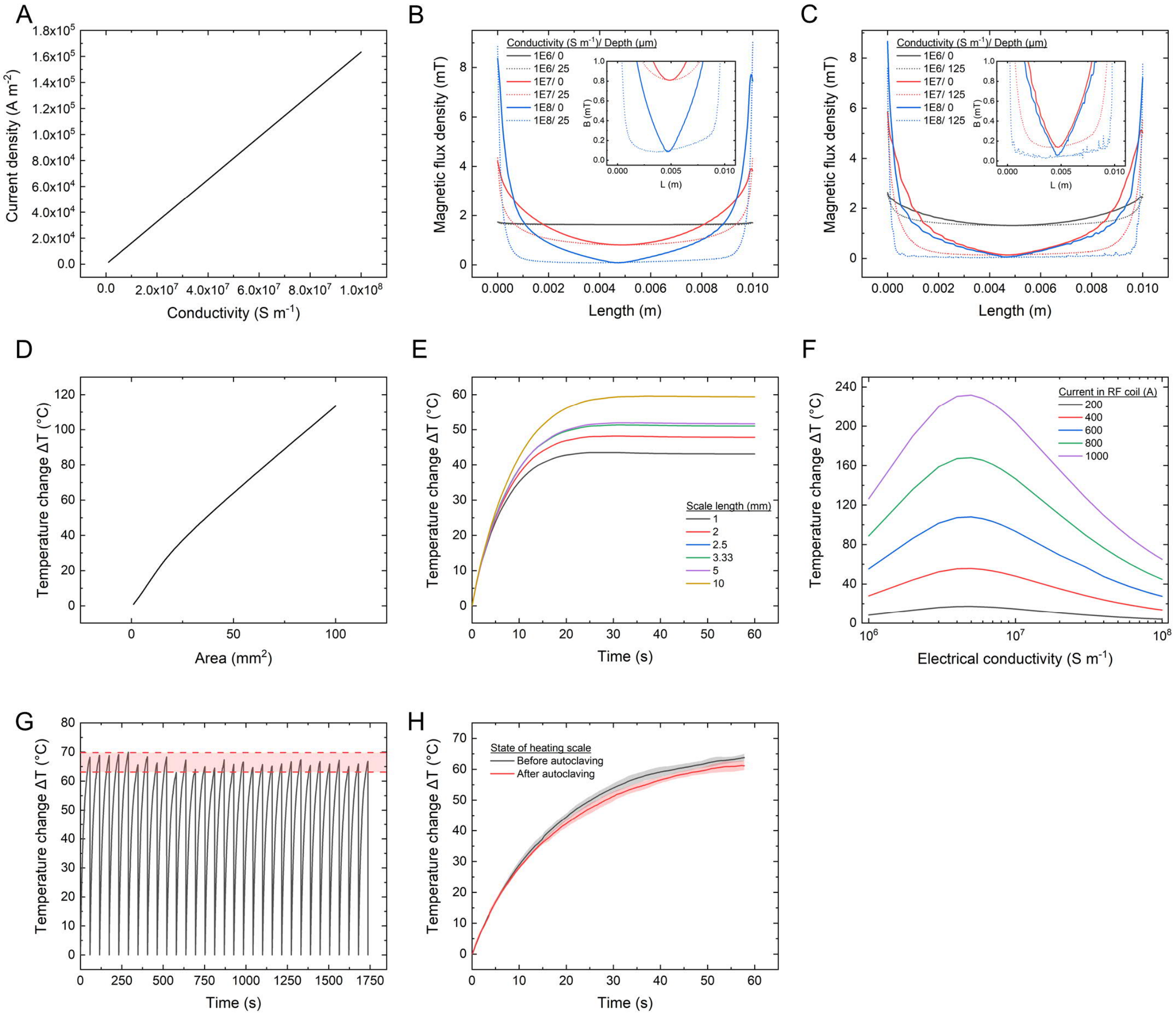

Supplementary Figure 2. Characterisation of heating performance with simulations in COMSOL. (**A**) Simulated current density against electrical conductivity. (**B**) Simulated magnetic flux density at different depths inside a 50 µm aluminium scale. (**C**) Simulated magnetic flux density at different depths inside a 250 µm aluminium scale. (**D**) Simulated temperature change of a 100 µm aluminium scale with different areas. (**E**) Simulated temperature change of 100 µm aluminium scales with identical areas but composed of different scale lengths over time. (**F**) Simulated temperature change of a 100 µm aluminium scale when different currents are applied in the RF coil at t = 60 s. (**G**) Temperature change of 16 non-overlapping 2.5 mm 100 µm-thick aluminium scales over 30 heating and cooling cycles. Region demarcated in red indicates the temperatures that fall within 5% of the average maximum value. (**H**) Temperature change of 16 non-overlapping 2.5 mm 100 µm aluminium scales before and after autoclaving (n = 5).

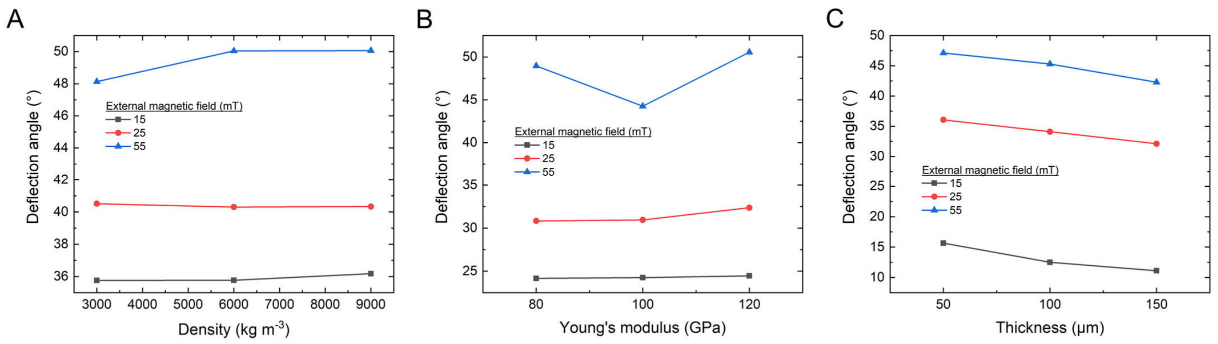

Supplementary Figure 3. Simulated deflection angles in COMSOL showing that the deflection angles are independent of the identified material properties. (**A**) Simulated deflection angles at different densities for a scale length of 2.5 mm of thickness 50 μm at a constant Young's modulus of 80 GPa. (**B**) Simulated deflection angles at different Young's moduli for a scale length of 2.5 mm of thickness 50 μm at a constant density of 6000 kg m^{-3}. (**C**) Simulated deflection angles at different thicknesses for a scale length of 2.5 mm at a constant density of 6000 kg/m^3 and constant Young's modulus of 100 GPa.

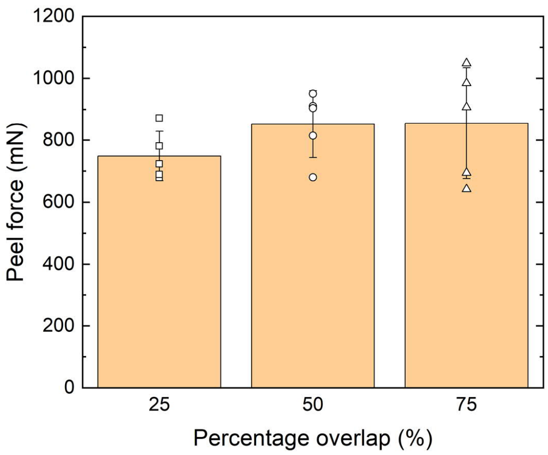

Supplementary Figure 4. Pull-out force of a 100 μm-thick aluminium scale from mPDMS at different percentage overlaps (n = 5).

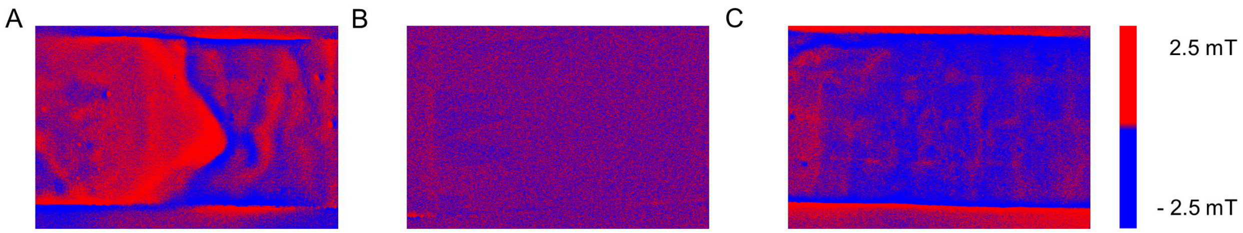

Supplementary Figure 5. Imaging of the magnetic fields generated by the soft magnetic polymer at various stages of actuation. (**A**) Before RF heating. (**B**) After RF heating. (**C**) After re-magnetisation. Outlines of the heating plates can be seen.

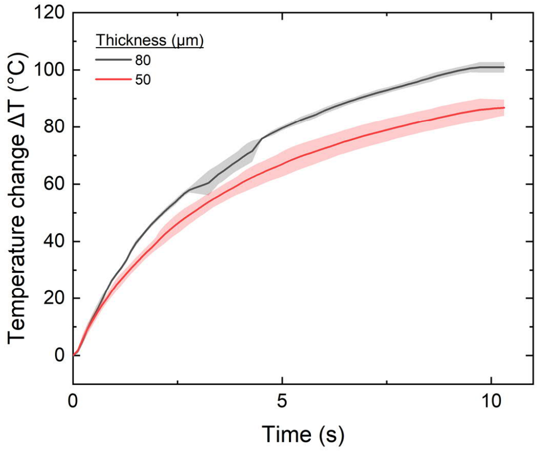

Supplementary Figure 6. Heating performance of a square 2.5 mm aluminium plate of different thicknesses placed 3 cm away from the RF coil (n = 3).

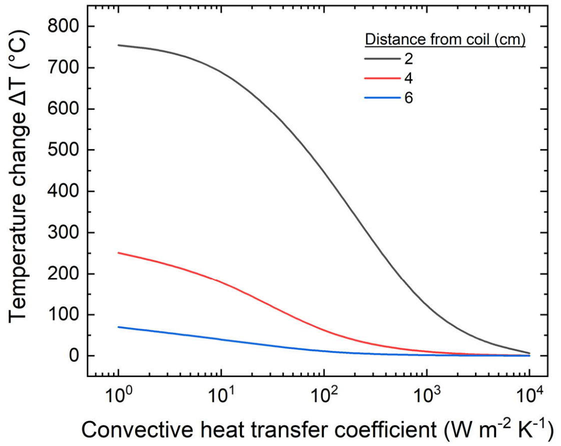

Supplementary Figure 7. Simulated temperatures of a 100 μm aluminium scale at t = 60 s at different convective heat transfer coefficients and distances from the RF coil.

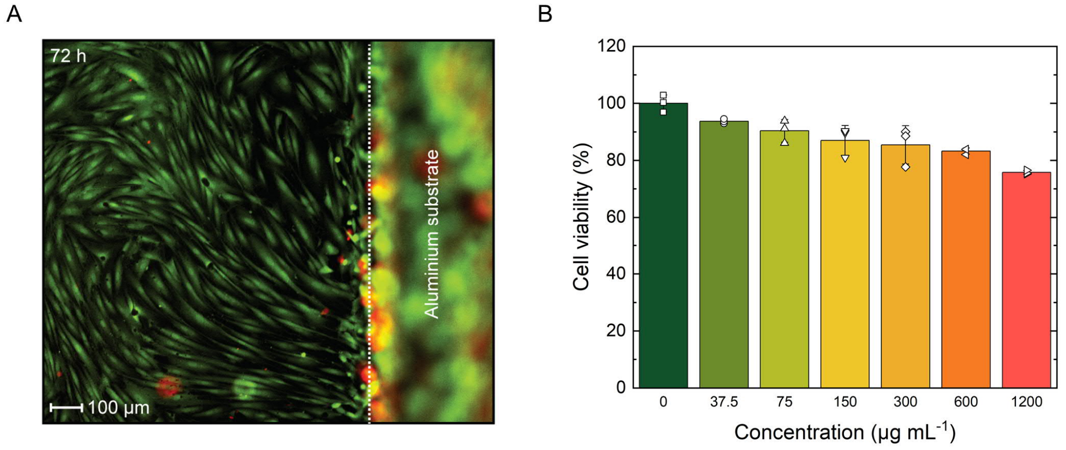

Supplementary Figure 8. Results from the initial biocompatibility tests. (A) Live-dead staining of fibroblast cells cultured with the aluminium scale. The cells were viable after 72 hours of culture and displayed healthy morphology even at the interface of the scale. **(B)** Cell viability as a function of aluminium powder concentration after 72 hours of treatment, based on adenosine triphosphate (ATP) production. The cells showed more than 85% viability even at relatively high concentrations over 3 independent samples (n = 3).

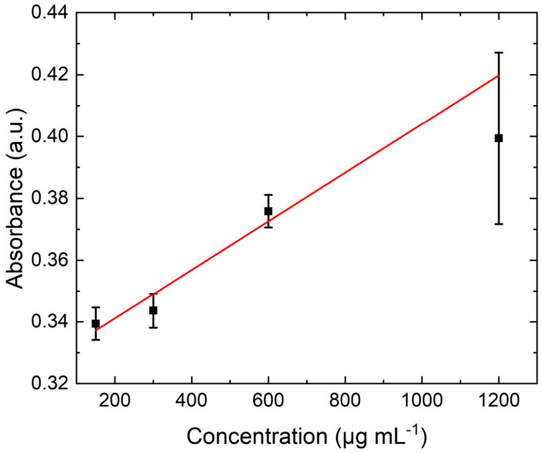

Supplementary Figure 9. Absorbance calibration curve for aluminium powder of varying concentrations suspended in simulated gastric fluid at 300 nm. 3 independent samples were tested once each (n = 3). Red line indicates the line of best fit.

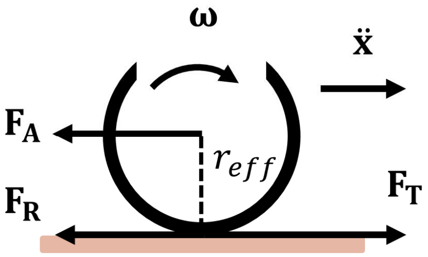

Supplementary Figure 10. Free body diagram of the rolling robot.

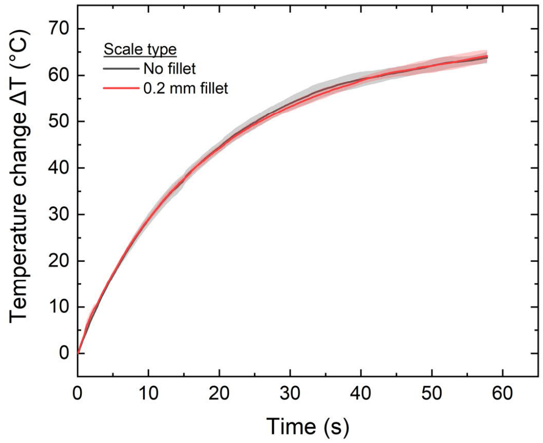

Supplementary Figure 11. Heating performance of 16 non-overlapping 2.5 mm square 100 μm aluminium scales with 0.2 mm fillets as compared to another with identical dimensions without fillets (n = 6).

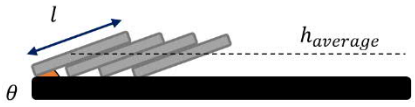

$$\theta = \tan^{-1}\frac{w}{l \times (1 - percentage\ overlap)}$$

$$h_{average} = \frac{1}{2}\sin\theta$$

Supplementary Figure 12. Calculation of the average height for calculation of the stress and strain for overlapping scales.

Supplementary Table 1. Summary of the various parameters which can be controlled to optimise the heating performance of a single scale.

Control parameter	Classification	Effects	Considerations
L	Geometrical	Increasing L increases final temperature	$\frac{1}{\delta xy}$ must be kept constant<br><br>Mechanical compliance
w	Geometrical	Increasing w decreases rate of temperature rise	$\frac{1}{\delta xy}$ must be kept constant<br><br>Mechanical compliance
σ	Material	Increasing σ decreases w	$\frac{1}{\delta xy}$ must be kept constant
ρ	Material	Decreasing ρ increases rate of temperature rise	Mechanical compliance
c_p	Material	Decreasing c_p increases rate of temperature rise	-
f	RF field	Increasing f increases final temperature	$\frac{1}{\delta xy}$ must be kept constant
I_{in}	RF field	Increasing I_{in} increases final temperature	-

Supplementary Table 2. Comparison of the RF fields applied in vivo in literature.

Study type	Frequency (kHz)	Maximum magnetic field intensity (kA/m)	Duration (min)	Reference
In vitro	118	30.6	30	(*9*)
Rats	118	30.6	30	(*10*)
Rats	118	30.6	30	(*11*)
Rats	118	30.6	30	(*12*)
Rats	100	18	40	(*13*)
Rats	150	11	20	(*14*)
Clinical trial	100	18	60	(*15*)
Clinical trial	100	15	60	(*16*)
Clinical trial	100	18	60	(*17*)
Clinical trial	100	18	60	(*18*)
This work	338	34.6	15	-

Codes: Data Processing

three_pt_bending_processing.m

```
 % % Test variables
L = 10; % support span [mm]
h = 0.2 + 0.0499600479360895; % thickness of beam including plate thickness [mm]
b = 10; % width of beam [mm]

% % INPUT FILE LOCATION
A{1} = readmatrix('C:\Users\Desktop\1 (1).csv');
A{2} = readmatrix('C:\Users\Desktop\1 (2).csv');
A{3} = readmatrix('C:\Users\Desktop\1 (3).csv');

force_combin = zeros(15000,3);
mid_span_deflection = zeros(15000,3);

for n = 1:3
    T = A{n};
    for i = 1:15000
    force_combin(i,n) = T(i,3);
    mid_span_deflection(i,n) = T(i,2);
    end
end

 % % GET AVERAGE AND STD
stress = zeros(15000,3);
strain = zeros(15000,1);
export = zeros(15000,3);

for j = 1:3
    stress (:,j) = force_combin(:,j) * 10^-3 * 1.5 * L / (b * h^2);
    strain (:,j) = mid_span_deflection(:,j) * 6 * h / L^2;
```

```
end
export(:,1) = mean(strain,2);
export(:,2) = mean(stress,2);
export(:,3) = std(stress,[],2);
```

<u>temperature_data_processing_batch.m</u>

```
% % INPUT FRAME SIZE AND FILE LOCATION
frame_size = 60;

A{1} = readmatrix('C:\Users\Desktop\1 (1).csv');
A{2} = readmatrix('C:\Users\Desktop\1 (2).csv');
A{3} = readmatrix('C:\Users\Desktop\1 (3).csv');
A{4} = readmatrix('C:\Users\Desktop\1 (4).csv');
A{5} = readmatrix('C:\Users\Desktop\1 (5).csv');
A{6} = readmatrix('C:\Users\Desktop\1 (6).csv');

temp_combin = zeros(500,6);

for n = 1:6
    T = A{n};
    rows = size(T,1);
    columns = size(T,2);
    frames = rows/ frame_size;
    B = zeros(rows/2, columns/2);
    C = zeros(frames,1);
    temp = zeros(frames,1);
    temp_1 = zeros(frames+1,1);
    temp_final = zeros(frames,1);

    % % CONVERT TEMPERATURE
    for j = 1:rows
        for k = 1:columns/2
```

```
        B(j,k) = T(j,2*k)+ T(j,(2*k))/ 1000;
    end
end
% % GET AVERAGE
for i = 1:frames
    for j = 1:frame_size
        for k = 1:frame_size
        C(i) = C(i) + B(j+ frame_size*(i-1),k);
        end
    end
end
C = C/ frame_size^2;
temp = C - C(1,1);
i = 2;

% % REMOVE INITIAL ZERO-PORTION
for j = 1:frames
    if temp (j,1) >= 0.1
        temp_1 (i,1) = temp (j,1);
        i = i + 1;
 end
end

% % REMOVE RF OFF
for i = 1:frames
    if temp_1 (i+1,1) - temp_1 (i,1) <= 0 && i > 500
        temp_1 (i,1) = 0;
    end
end

% % REMOVE NOISE
    temp_final = medfilt1 (temp_1,20);
```

```
    % % Remove last digits from filtered results
        for i = 1:500
            temp_combin(i,n) = temp_final(i,1);
        end
end

% % Final
temp_process = zeros (500,2);
for i = 1:500
   temp_process(:,1) = mean(temp_combin,2);
   temp_process(:,2) = std(temp_combin,[],2);
 end
```

Supplementary References

1. V. Rudnev, D. Loveless, R. L. Cook, M. Black, *Handbook of Induction Heating* (CRC Press, 2002; https://www.taylorfrancis.com/books/9781420028904).
2. D. J. Griffiths, *Introduction to Electrodynamics* (Cambridge University Press, 2017; https://www.cambridge.org/highereducation/product/9781108333511/book).
3. Jack P. Holman, *Heat Transfer* (McGraw-Hill, New York, Tenth Edit., 2010).
4. W. Hu, G. Z. Lum, M. Mastrangeli, M. Sitti, Small-scale soft-bodied robot with multimodal locomotion. *Nature*. **554**, 81–85 (2018).
5. P. A. Hasgall, F. Di Gennaro, C. Baumgartner, E. Neufeld, B. Lloyd, M. C. Gosselin, D. Payne, A. Klingenböck, N. Kuster, IT'IS Database for Thermal and Electromagnetic Parameters of Biological Tissues (2022), , doi:10.13099/VIP21000-04-1.
6. S. K. . Lai, Y.-Y. Wang, D. Wirtz, J. Hanes, Micro- and macrorheology of mucus. *Adv. Drug Deliv. Rev.* **61**, 86–100 (2009).
7. R. K. Finn, Determination of the Drag on a Cylinder at Low Reynolds Numbers. *J. Appl. Phys.* **24**, 771–773 (1953).
8. J. C. Páscoa, F. P. Brójo, F. C. Santos, P. O. Fael, An innovative experimental on-road testing method and its demonstration on a prototype vehicle. *J. Mech. Sci. Technol.* **26**, 1663–1670 (2012).
9. M. Shinkai, M. Yanase, H. Honda, T. Wakabayashi, J. Yoshida, T. Kobayashi, Intracellular Hyperthermia for Cancer Using Magnetite Cationic Liposomes: In vitro Study. *Japanese J. Cancer Res.* **87**, 1179–1183 (1996).
10. M. Yanase, M. Shinkai, H. Honda, T. Wakabayashi, J. Yoshida, T. Kobayashi, Antitumor Immunity Induction by Intracellular Hyperthermia Using Magnetite Cationic Liposomes. *Japanese J. Cancer Res.* **89**, 775–782 (1998).
11. A. Ito, M. Shinkai, H. Honda, K. Yoshikawa, S. Saga, T. Wakabayashi, J. Yoshida, T. Kobayashi, Heat shock protein 70 expression induces antitumor immunity during intracellular hyperthermia using magnetite nanoparticles. *Cancer Immunol. Immunother.* **52**, 80–88 (2003).
12. B. Le, M. Shinkai, T. Kitade, H. Honda, J. Yoshida, T. Wakabayashi, T. Kobayashi, Preparation of Tumor-Specific Magnetoliposomes and Their Application for Hyperthermia. *J. Chem. Eng. JAPAN*. **34**, 66–72 (2001).
13. A. Jordan, R. Scholz, K. Maier-Hauff, F. K. H. van Landeghem, N. Waldoefner, U. Teichgraeber, J. Pinkernelle, H. Bruhn, F. Neumann, B. Thiesen, A. von Deimling, R. Felix, The effect of thermotherapy using magnetic nanoparticles on rat malignant glioma. *J. Neurooncol.* **78**, 7–14 (2006).
14. I. Rabias, D. Tsitrouli, E. Karakosta, T. Kehagias, G. Diamantopoulos, M. Fardis, D. Stamopoulos, T. G. Maris, P. Falaras, N. Zouridakis, N. Diamantis, G. Panayotou, D. A. Verganelakis, G. I. Drossopoulou, E. C. Tsilibari, G. Papavassiliou, Rapid magnetic heating treatment by highly charged maghemite nanoparticles on Wistar rats exocranial glioma tumors at microliter volume. *Biomicrofluidics*. **4**, 1–8 (2010).
15. K. Maier-Hauff, R. Rothe, R. Scholz, U. Gneveckow, P. Wust, B. Thiesen, A. Feussner, A. Deimling, N. Waldoefner, R. Felix, A. Jordan, Intracranial thermotherapy using magnetic nanoparticles combined with external beam radiotherapy: Results of a feasibility study on patients with glioblastoma multiforme. *J. Neurooncol.* **81**, 53–60 (2007).
16. K. Maier-Hauff, F. Ulrich, D. Nestler, H. Niehoff, P. Wust, B. Thiesen, H. Orawa, V. Budach, A. Jordan, Efficacy and safety of intratumoral thermotherapy using magnetic iron-oxide nanoparticles combined with external beam radiotherapy on patients with recurrent glioblastoma multiforme. *J. Neurooncol.* **103**, 317–324 (2011).
17. ClinicalTrials.gov, Study of Focal Ablation of the Prostate With NanoTherm® Therapy System for Intermediate-Risk Prostate CancerNo Title (2022), (available at https://clinicaltrials.gov/ct2/show/study/NCT05010759).
18. F. K. H. van Landeghem, K. Maier-Hauff, A. Jordan, K. T. Hoffmann, U. Gneveckow, R. Scholz, B. Thiesen, W. Brück, A. von Deimling, Post-mortem studies in glioblastoma patients treated with thermotherapy using magnetic nanoparticles. *Biomaterials*. **30**, 52–57 (2009).
