# Supplementary material for: Pangolin-inspired untethered magnetic robot for on-demand biomedical heating applications
Source: Nat Commun. 2023 Jun 20;14:3320. doi: 10.1038/s41467-023-38689-x (PMC10282021; doi:10.1038/s41467-023-38689-x)
Supplement: Supplementary file 3 — Description of Additional Supplementary Files [file 41467_2023_38689_MOESM3_ESM.pdf]

Description of Additional Supplementary Files

Supplementary Movie 1. Magnetic actuation of robot in stomach phantom – 100 µm-thick aluminium scales @ 50 % overlap with a 65 mT rotating field.

Supplementary Movie 2. In situ demagnetisation enables different locomotion modes.

Supplementary Movie 3. Selective cargo release.

Supplementary Movie 4. Robot with thermal adhesive.

Supplementary Movie 5. Application of heat to a wound mitigates blood loss.

Supplementary Movie 6. Ultrasound guided robot in ex-vivo porcine small intestine.
