# Supplementary material for: Pangolin-inspired untethered magnetic robot for on-demand biomedical heating applications
Source: Nat Commun. 2023 Jun 20;14:3320. doi: 10.1038/s41467-023-38689-x (PMC10282021; doi:10.1038/s41467-023-38689-x)
Supplement: Supplementary file 10 — Reporting Summary [file 41467_2023_38689_MOESM10_ESM.pdf]

nature portfolio

Corresponding author(s): Metin Sitti

Last updated by author(s): Apr 25, 2023

Reporting Summary

Nature Portfolio wishes to improve the reproducibility of the work that we publish. This form provides structure for consistency and transparency in reporting. For further information on Nature Portfolio policies, see our Editorial Policies and the Editorial Policy Checklist.

Statistics

For all statistical analyses, confirm that the following items are present in the figure legend, table legend, main text, or Methods section.

n/a	Confirmed	
☐	☒	The exact sample size (*n*) for each experimental group/condition, given as a discrete number and unit of measurement
☐	☒	A statement on whether measurements were taken from distinct samples or whether the same sample was measured repeatedly
☒	☐	The statistical test(s) used AND whether they are one- or two-sided<br>*Only common tests should be described solely by name; describe more complex techniques in the Methods section.*
☒	☐	A description of all covariates tested
☒	☐	A description of any assumptions or corrections, such as tests of normality and adjustment for multiple comparisons
☐	☒	A full description of the statistical parameters including central tendency (e.g. means) or other basic estimates (e.g. regression coefficient) AND variation (e.g. standard deviation) or associated estimates of uncertainty (e.g. confidence intervals)
☒	☐	For null hypothesis testing, the test statistic (e.g. *F*, *t*, *r*) with confidence intervals, effect sizes, degrees of freedom and *P* value noted<br>*Give P values as exact values whenever suitable.*
☒	☐	For Bayesian analysis, information on the choice of priors and Markov chain Monte Carlo settings
☒	☐	For hierarchical and complex designs, identification of the appropriate level for tests and full reporting of outcomes
☒	☐	Estimates of effect sizes (e.g. Cohen's *d*, Pearson's *r*), indicating how they were calculated

Our web collection on statistics for biologists contains articles on many of the points above.

Software and code

Policy information about availability of computer code

Data collection	FLIR Thermal Studio, COMSOL 6.0<br>Heating characterisation experiments were performed on three separate samples tested twice each.<br>Mechanical characterisation experiments were performed on three separate samples tested once each.
Data analysis	ImageJ 1.52p, MATLAB R2019A, Origin 2019b

For manuscripts utilizing custom algorithms or software that are central to the research but not yet described in published literature, software must be made available to editors and reviewers. We strongly encourage code deposition in a community repository (e.g. GitHub). See the Nature Portfolio guidelines for submitting code & software for further information.

Data

Policy information about availability of data

All manuscripts must include a data availability statement. This statement should provide the following information, where applicable:

- Accession codes, unique identifiers, or web links for publicly available datasets
- A description of any restrictions on data availability
- For clinical datasets or third party data, please ensure that the statement adheres to our policy

Source data are provided with this paper.

Human research participants

Policy information about studies involving human research participants and Sex and Gender in Research.

Reporting on sex and gender	NA
Population characteristics	NA
Recruitment	NA
Ethics oversight	NA

Note that full information on the approval of the study protocol must also be provided in the manuscript.

Field-specific reporting

Please select the one below that is the best fit for your research. If you are not sure, read the appropriate sections before making your selection.

☒ Life sciences ☐ Behavioural & social sciences ☐ Ecological, evolutionary & environmental sciences

For a reference copy of the document with all sections, see nature.com/documents/nr-reporting-summary-flat.pdf

Life sciences study design

All studies must disclose on these points even when the disclosure is negative.

Sample size	All biological measurements and experiments were performed in triplicates. 3 samples were used in both the biocompatibility and hyperthermia studies to account for any variabilities across the studies.
Data exclusions	No data were excluded from the analyses.
Replication	At least three replicates per group. All attempts at replication were successful.
Randomization	In the biocompatibility study, the fibroblasts were cultured on aluminium substrates that were selected at random. 6 samples were prepared and 3 were selected at random. Aluminium solutions of varying concentrations were added sequentially to 96 wells with fibroblast cells seeded the day before to determine the cell viability as a function of the concentration.<br>In the hyperthemia study, the tumour spheriods were selected at random after culture for testing.
Blinding	As the outcomes of the experiments were clearly and objectively defined (luminescence values were measured using a plate reader), blinding was not relevant.

Reporting for specific materials, systems and methods

We require information from authors about some types of materials, experimental systems and methods used in many studies. Here, indicate whether each material, system or method listed is relevant to your study. If you are not sure if a list item applies to your research, read the appropriate section before selecting a response.

Materials & experimental systems

n/a	Involved in the study	
☒	☐	Antibodies
☐	☒	Eukaryotic cell lines
☒	☐	Palaeontology and archaeology
☒	☐	Animals and other organisms
☒	☐	Clinical data
☒	☐	Dual use research of concern

Methods

n/a	Involved in the study	
☒	☐	ChIP-seq
☒	☐	Flow cytometry
☒	☐	MRI-based neuroimaging

Eukaryotic cell lines

Policy information about cell lines and Sex and Gender in Research

Cell line source(s)	Human healthy fibroblast cells, CRL-2522 (ATCC) and Epithelial colon tumour cells, HT-29 (ATCC)

Authentication	Cell line was not authenticated.
Mycoplasma contamination	Negative
Commonly misidentified lines (See ICLAC register)	No commonly misidentified cell line was used in the study.
